# Exploring the genetic factors of nitrogen use efficiency in potato

**DOI:** 10.1371/journal.pone.0325578

**Published:** 2025-11-14

**Authors:** Miguel Ángel Mendoza-Bustamante, Aura Natalia Jiménez-Medrano, Johana Carolina Soto-Sedano, María Cecilia Delgado-Niño, Stanislav Magnitskiy, Gustavo Adolfo Ligarreto-Moreno, Teresa Mosquera-Vásquez

**Affiliations:** 1 Universidad Nacional de Colombia, Sede Bogotá, Facultad de Ciencias Agrarias, Bogotá, Colombia; 2 Universidad Nacional de Colombia, Sede Bogotá, Departamento de Biología, Facultad de Ciencias, Bogotá, Colombia; Osmania University, INDIA

## Abstract

Nitrogen is an essential nutrient for plants, used by farmers to increase the yield of crops. However, this practice increases greenhouse gases, negatively affecting the environment. Nitrogen Use Efficiency (NUE) is a trait that is beginning to be studied in some model species and in cereals due to its complex and novel trait nature. In potatoes, the information is limited. Therefore, this research can help to mitigate the environmental impact of nitrogen fertilizer use, reducing groundwater contamination and greenhouse gas emissions. The study of NUE at the genetic level, based on a diverse population in potato materials, will contribute to the understanding of the genetic architecture of the trait. This research evaluated NUE in a *Solanum tuberosum* diploid potato genetic diversity panel from the Phureja group. The characterization of the trait was carried out in substrate conditions, for low and high levels of nitrogen for both the vegetative and the reproductive phase. Eighteen variables associated with NUE were measured, nine under low nitrogen and nine under high nitrogen conditions. A GWAS was conducted, and a total of 21 QTNs were identified as strongly associated with 11 phenotypic variables related to NUE in potato, these QTNs explained between 0.08% and 2.7% of the observed phenotypic variation, along with a repertoire of 750 candidate genes associated with the trait. This research aimed to establish the basis for understanding the genetic architecture of NUE in *Solanum tuberosum*. Once the candidate genes are validated, they could be used to develop molecular tools and accelerate breeding programs aimed at improving NUE in *Solanum tuberosum*.

## Introduction

Potato is an essential food for humanity, characterized by its high nutritional value, significant yield potential, and wide adaptability, making it a crucial crop for global food security [1]. It is considered an important source of carbohydrates, but it also provides dietary fiber, vitamin C, vitamin B6, potassium, magnesium, iron, carotenoids, and phenolic acids [2–5]. It is affordable food that is accessible throughout the year. The dominant approach in potato production has followed a linear model, assuming that higher inputs lead to higher yields [6].This model has resulted in the overuse of fertilizers, especially nitrogen (N), which has lowered efficiency and contributed to water pollution and soil degradation [6]. This approach mirrors the broader trends seen since the Green Revolution, which, following the end of World War II, introduced synthetic chemical pesticides and fertilizers [7]. These products led to increased crop yields, with wheat yields increasing by up to 400% [8,9].This positioned nitrogen as one of the most important fertilizers in crop production. In the last 20 years, there has not been a proportional increase in crop yields despite increased applications of nitrogen fertilizers [10]. It is reported that between 50% to 75% of the nitrogen applied as fertilizer is not utilized by plants, instead leaching as nitrates *(NO*_*3*_^—^) or volatilizing as ammonia *(NH*_*3*_) and nitrous oxide (*N*_*2*_*O)*, thus polluting the environment [11,12]. As a result, Nitrogen Use Efficiency (NUE) has become the second most studied trait worldwide due to its importance in agriculture for optimizing fertilization and its potential for breeding programs [13]. However, the genetic control of this trait remains largely unexplored, as NUE is a complex trait governed by multiple genes involved in control [14]. To reduce the use of nitrogen fertilizers in crop production, efforts have been made to decipher the architecture of the traits in crops such as maize, wheat, and rice [15–17].

Genome-wide association studies (GWAS) are a valuable strategy for identifying genomic regions associated with a trait of interest [18]. Using a naturally diverse population enhances the likelihood of identifying superior alleles in the gene pool and provides more precise mapping [19]. Genetic association studies in NUE have identified candidate genes such as *ZmNAC36* for maize [20], *NPF2.12* for wheat [15], and *OsNIGT1* for rice [12], among others. Understanding the architecture of the traits in rice has improved NUE by up to 40% with the overexpression of the *OsNRT2.3b* gene, enhancing the buffering capacity of the plant cell, to increase nitrogen uptake [21].

In the case of potato, NUE studies have mainly focused on improving cultural practices or soil management, except for some researchers who have conducted genetic studies using linkage mapping [22] and association mapping [13,23,24]. Although these studies have been valuable, the genetic architecture underlying NUE still has much to be uncovered. This may be due to the methodologies used for phenotyping, as the studies were conducted under field conditions where precipitation, temperature, and their interaction with soils rich in minerals and microorganisms affect nitrogen availability, thus influencing the proposed treatments [25,26]. It may also be explained by the genetic diversity panel of European populations used in these studies, as well as by the contrasting precocity of the genotypes [13,22,24]. Therefore, it is necessary to conduct studies with highly diverse populations that allow the study of natural allelic diversity, with phenotyping in controlled environments that establish conditions of nitrogen deficiency and sufficiency, to help decipher the genetic architecture of the trait, generating knowledge about the genetic architecture of NUE in a diversity panel of diploid potato through genetic association analysis.

In this context, this study aimed to identify genetic variants associated with NUE in a potato population using GWAS, with the goal of contributing to genetic improvements in potato for optimized NUE. To achieve this, NUE was evaluated in a diploid population from the Phureja group, selected for its genetic and nutritional potential [27,28], making it an excellent candidate for investigating genetic variability and enhancing this trait. The QTNs (Quantitative Trait Nucleotides) and candidate genes identified potentially associated with NUE, enhancing our fundamental understanding of the genetic mechanisms underlying the NUE, which could guide future genetic breeding strategies to optimize this trait in potatoes.

## Materials and methods

### Plant material and essay conditions

A total of 100 accessions (genotypes) of diploid potato, *Solanum tuberosum* Phureja group, from the Working Collection of the Potato Breeding Program of the Universidad Nacional de Colombia, plus five diploid commercial varieties: Criolla Colombia, Criolla Galeras, Criolla Guaneña, Criolla Latina, Criolla Paisa, were used as association panel (S1 Table). This group is known for its lack of tuber dormancy [29,30] and also was used to assemble the reference potato genome [31]. The trial was established in the greenhouses of the Faculty of Agricultural Sciences of the Universidad Nacional de Colombia (4°38’16.7 “N, 74°05’18.2” W). During the crop cycle the average air temperature was 19.3 °C, with a maximum and the minimum temperature of 38.8°C and 8°C respectively. The average relative air humidity was 63.6% and the average dew point was 11.38°C.

The trial was carried out under a completely randomized design (CRD), with 2 treatments and 3 biological experimental units (plants) per genotype and treatment. The plants were grown in plastic bags with a 4 kg substrate capacity. The substrate used was a mixture (v/v) of 70% peat (Klasmann^®^ No. 413 peat, 0–5 mm, without added nutrients) and 30% perlite (8–12 mm). This system, as a Semi-aeroponic system, allows for controlled nutrient supply without affecting tuber development.

### Phenotyping Nitrogen Use Efficiency (NUE) parameters in potato

#### Establishment of nitrogen deficient and sufficient treatments.

Treatments (T) consisted of a low nitrogen dose (nitrogen-deficient conditions) and a sufficient nitrogen dose (high nitrogen conditions), applied at 2 crop stages: vegetative and reproductive stage. For the vegetative stage, the dose of low nitrogen was 19 ppm and 195 ppm for high nitrogen, while, for the reproductive stage, there was low nitrogen 15 ppm and high nitrogen 146 ppm. These doses were established based on the findings reported by [32], ensuring that the applied levels adequately reflect contrasting nitrogen conditions for the panel under study. Nitrogen was supplied as ammonium nitrate (NH_4_NO_3_) of analytical grade purity. Total nitrogen supply per plant per growth cycle, including nitrogen content in water and substrate, was 0.56 g (low nitrogen) and 1.78 g (high nitrogen). These values were calculated based on the volume of water supplied throughout the crop cycle and the nitrogen concentration applied, expressed in ppm.

The dosages of each macronutrient and micronutrient (in ppm), excluding nitrogen, were established for the vegetative and reproductive stages following the criteria reported by [32]: P (35, 22), K (200, 260), Ca (64, 64), S (40, 40), Mg (24, 24), B: (0.6, 0.6), Zn (0.2, 0.2), Cu (0.2, 0.2), Mn (0.75, 0.75), Fe (1.5, 1.5), Mo (0.04, 0.04). The nutrient solution was applied according to the water requirements of the crop, adjusting to the water needs of the plants and substrate moisture. In addition, the electrical conductivity of the substrate was monitored and maintained at approximately 1.5 dS ⋅ m^-1^. The volume of solutions applied to each plant was 8.6 L in total, 2.4 L in the vegetative stage and 6.4 L in the reproductive stage. The transition from the vegetative to the reproductive stage nutrient solution occurred 49 days after planting (DAP), corresponding to 556.1 growing degree days (GDD) calculated using the equation from [33].

#### Measurement of variables linked to nitrogen use efficiency.

A total of 18 phenotypic variables were measured per genotype across 3 biological experimental units, 9 under high N treatment and 9 under low N treatment. At 110 DAP, which corresponded to the time of harvest, the following variables were measured in each plant: 1) Relative chlorophyll content (SPAD74) at 74 days after planting (DAP) as an indicator of the N status of the plants with a portable Minolta SPAD 502® (Japan). Readings were taken in leaves as the median of 3 fully expanded leaves, in the upper third of each evaluated plant; 2) Number of stems (StemNo), 3) Stem length (StemL), was measured as the length of the longest stem on each plant, from the base where it joins the root system to the shoot apex; 4) Number of tubers (TubNo), were evaluated, as these traits have been previously associated with NUE in potato and other species through phenotyping and GWAS studies [6,23,34].

Samples of the aerial organs and tubers were collected separately and dried in an oven at 60 °C for 3–5 days until a constant weight was reached. The dry weight of the aerial part and the tubers were determined individually, and their sum was used to calculate the total dry weight. Also, samples of aerial organs (leaves and stems) were analyzed for Carbon percentage of the aerial part of the plant (%CAp) and tubers were analyzed for total nitrogen percentage (%Ntub), both using DUMAS This methodology is known to be simpler and faster and generates no chemical waste, using the Leco Fp828 mc 50282^®^ Series Elemental Analyzer [35,36]. These analyses were conducted at the Water and Soil Laboratory of the Faculty of Agricultural Sciences, Universidad Nacional de Colombia, Bogotá. Finally, the NUE indices described by [37] and [38] were calculated as follows: Nitrogen Use Efficiency (NUE, g. g^−1^): dry weight of tuber/N supplied. Agronomic Nitrogen Use Efficiency (AgNUE, g. g^−1^): Fresh weight of tubers/N supplied. Harvest Index (HI, g. g^−1^): dry weight of tubers/dry weight of total plant.

Phenotypic data were subjected to analysis of variance (ANOVA) within and between genotypes, followed by the Tukey’s test. To explore multivariate patterns within the phenotypic dataset and identify traits most strongly associated with NUE), a Principal Component Analysis (PCA) was performed using scaled phenotypic data collected from potato genotypes evaluated under contrasting nitrogen supply conditions. The analysis was conducted in R, using the FactoMineR [39] and factoextra [40] packages. PCA enabled dimensionality reduction and visualization of genotype distribution across multiple traits related to NUE performance.

To identify genotypes with contrasting Agronomic Nitrogen Use Efficiency (AgNUE), AgNUE values calculated under both high and low nitrogen conditions were plotted, with each point representing a genotype. A color gradient was used to indicate the percentage of nitrogen accumulated in tubers under low nitrogen conditions (%NutLowN). To further explore the relationship between yield and the carbon-to-nitrogen (C/N) balance, total nitrogen and carbon accumulation values under low nitrogen conditions were also plotted, with AgNUE represented through a continuous color scale. These visualizations provide insights into how nitrogen efficiency relates to biomass composition and source–sink dynamics under nitrogen-limited conditions. All plots were generated using the *ggplot2* package in R [41].

### Genotyping and genome-wide association study

We use genotyping by sequencing (GBS) SNP (single nucleotide polymorphism) data obtained by [42]. A new SNP calling was performed based on the latest potato reference genome V6.1 version [43], and the resulting SNP matrix is provided in S2 Table. For this, the quality of the reads (generated using the Illumina HiSeq2500 system) was assessed with the FastQC tool [44]. Subsequently, the reads with a Quality Score (QS) below 30 were cleaned using the Trimmomatic, and SNP calling was performed with the NGSEP [45] and TASSEL software [46]. Filtering SNPs with a frequency below 1% and those with missing data less than 15%, resulting in a matrix of 26,515 SNPs. From the VCF file and using the TASSEL software [46], were filtered those SNPs with minor allele frequency (MAF) ≥ 0.01 and present in at least 85% of the genotypes.

GWAS was performed using the mean values of the 18 phenotypic variables of NUE and the genotyping SNP matrix of 17,374 markers. The associations were conducted using the BLINK (Bayesian Linear Mixed Model) [47], MLMM (Mixed Linear Model Method) [48] and FarmCPU (Fixed and Random Model Circulating Probability Unification) [49] models implemented in the R package ‘Genomic Association and Prediction Integrated Tool’ (GAPIT) [50]. Principal components analysis (PCA) for population structure was established and incorporated as correction in the association models. Pairwise correlation coefficients were calculated, and the Linkage Disequilibrium (LD) mean decay was established by plotting the R^2^ values against the physical distance and visualizing it using TASSEL [46]. The significance threshold for detecting associated QTNs was set at *P* ≤ 0.05, with multiple-testing correction using the Benjamini–Hochberg (B&H) false discovery rate (FDR) procedure at ≤ 0.1 to balance discovery power with control of type I errors [51]. This correction was implemented through the GAPIT package V3 [50]. We selected an FDR cutoff of 0.1 because NUE is a highly complex trait likely governed by polygenic architectures with small to moderate effect sizes, for which overly stringent corrections may lead to the exclusion of biologically meaningful signals. The identified QTNs were visualized with Manhattan plots, and the model adjustments were established by quantile–quantile plots (QQ plots).

### Finding candidate genes associated with nitrogen use efficiency

The genomic positions of the identified QTNs were established at the latest potato reference genome V6.1 version [43]. Also, whether these regions correspond to intergenic, coding, intronic, or regulatory sequences. For those QTNs located within coding regions, it was determined whether the SNP causes a synonymous or nonsynonymous change in the gene’s codon. Also, the upstream and downstream genomic regions of each QTN were explored, considering the LD decay value in the association panel, to identify candidate genes for NUE in potato. Finally, the functional annotation of the candidate genes was consulted in the SpudBase for potato genome version v6.1, which was generated using data from the Arabidopsis proteome [52], the PFAM database v32 [53], and the Swiss Prot plant protein database [54].

## Results

### Significant differences in the phenotypic variables related to nitrogen use efficiency

**Fig 1:**
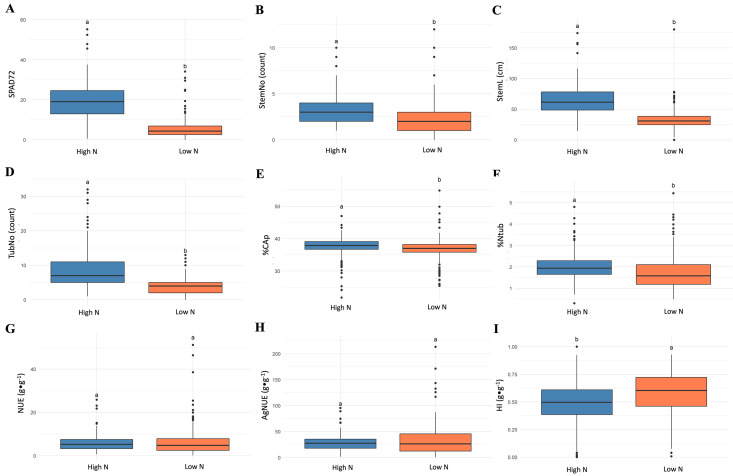
Behavior of phenotypic variables measured for treatments of high and low nitrogen in the potato association panel. **A.** Chlorophyll Relative Content (SPAD74). **B.** Number of stems (StemNo). **C.** Stem length (StemL). **D.** Number of tubers (TubNo). **E.** Carbon percentage of the aerial part of the plant (%CAp). **F.** Tuber N percentage (%Ntub). Nitrogen use efficiency indices (NUE): **G.** Nitrogen Use Efficiency (NUE, g ⋅ g − 1). **H.** Agronomic Nitrogen Use Efficiency (AgNUE, g ⋅ g − 1). **I.** Harvest Index (HI, g ⋅ g − 1). High nitrogen treatment in orange, low nitrogen treatment in blue. The bars indicate the standard deviation (SD), and different letters above each box indicate significant differences according to Tukey’s test (p < 0.05).

The genotypes in the association panel showed significant differences in the 18 phenotypic variables related to NUE measured under both high nitrogen and low nitrogen treatments (ANOVA, p < 0.01) (S3 Table). The variable SPAD74 values ranged from 13.6 to 48.9 under high nitrogen, with an average of 33.93, while under low nitrogen, values varied between 14.1 and 41.9, with a mean of 27.51, representing an 18.92% decrease (Fig 1A).

Regarding the variable StemNo, this ranged from 1 to 10 under high nitrogen, averaging 2.97, whereas under low nitrogen, values spanned from 1 to 12, with a mean of 2.55, reflecting a 14.14% reduction (Fig 1B). StemL exhibited a broad range, from 14.4 to 174 cm under high nitrogen, with an average of 63.84 cm. Under low nitrogen, values ranged from 4.3 to 180 cm, with a mean of 32.73 cm, indicating a 48.73% decrease (Fig 1C). A similar pattern was observed for TubNo, which varied from 1 to 32 under high nitrogen, averaging 8.57. Under low nitrogen values decreased substantially, ranging from 0 to 13, with a mean of 4.18, corresponding to a 51.22% reduction (Fig 1D).

The organ analysis showed that %CAp under high N ranged from 21.8 to 47%, with an average of 37.63%. Under low nitrogen, values varied between 25.4 and 54.9%, with a mean of 36.9%, showing a 1.94% decline (Fig 1E). The variable %Ntub fluctuated from 0.3 to 4.8% under high nitrogen, with a mean of 2.01%, whereas under low nitrogen, values ranged from 0.49 to 5.44%, averaging 1.72%, resulting in a 14.43% decrease (Fig 1F). The evaluation of NUE indices showed a different trend. NUE ranged from 0.61 to 25.87 g ⋅ g ⁻ ¹ under high nitrogen, with a mean of 5.8 g ⋅ g ⁻ ¹. Under low nitrogen, it varied from 0.18 to 51.15 g ⋅ g ⁻ ¹, with an average of 6.32 g ⋅ g ⁻ ¹, marking an 8.97% increase (Fig 1G). A similar trend was observed for AgNUE, which ranged from 3.37 to 102.67 g ⋅ g ⁻ ¹ under high nitrogen, averaging 29.36 g ⋅ g ⁻ ¹. Under low nitrogen, values were higher, spanning from 0.52 to 213.29 g ⋅ g ⁻ ¹, with a mean of 32.23 g ⋅ g ⁻ ¹, indicating a 9.78% increase (Fig 1H). Finally, HI followed an opposite trend, ranging from 0 to 1 g ⋅ g ⁻ ¹ under high nitrogen, with an average of 0.49 g ⋅ g ⁻ ¹. Under low nitrogen, values were slightly higher, varying from 0.01 to 0.93 g ⋅ g ⁻ ¹, with a mean of 0.58 g ⋅ g ⁻ ¹, reflecting a 19.75% increase (Fig 1I).

**Fig 2:**
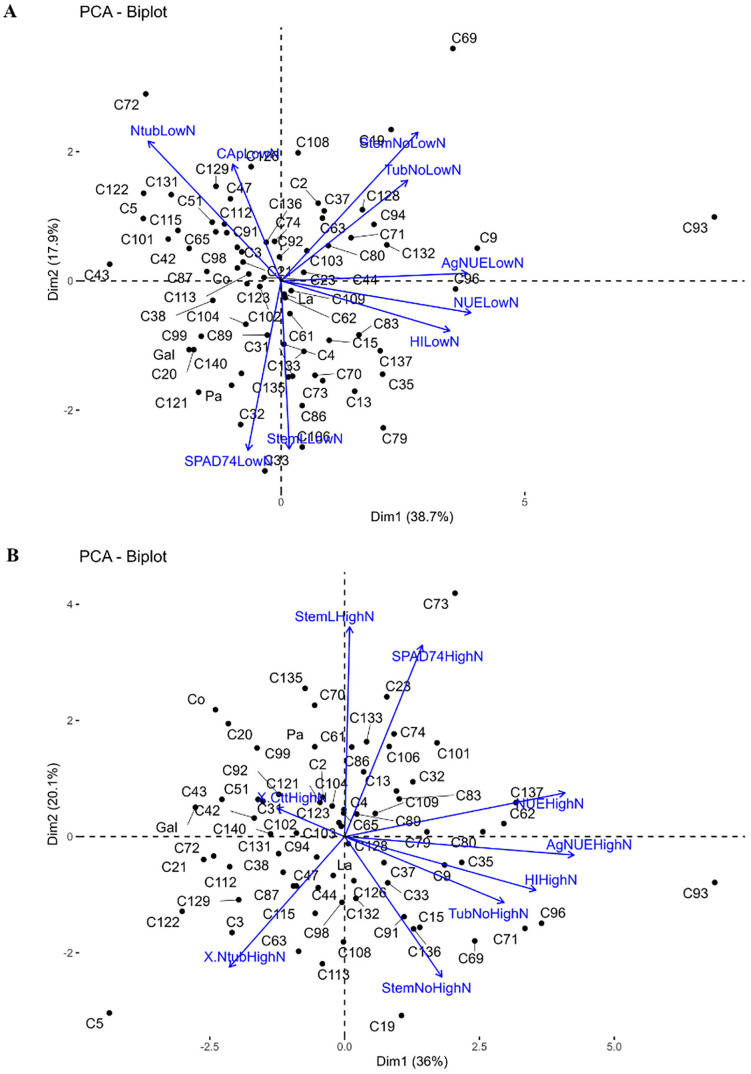
Principal Component Analysis (PCA) of phenotypic variables related to nitrogen use efficiency in the potato association panel. The PCA illustrates the distribution of the phenotypic variables across the first two principal components for **A**. Treatment of low nitrogen. **B**. Treatment of high nitrogen.

The PCA shows that under both low and high nitrogen conditions, the first two components explained 56.6% (Fig 2A: Dim1 = 38.7%, Dim2 = 17.9%) and 55.6% (Fig 2B: Dim1 = 35.2%, Dim2 = 20.4%) of the total variation in the phenotypic variables, respectively (S4, S5 Tables). The PCA shows that variables such as AgNUE, NUE, HI, TubNo, and StemNo are highly correlated in both treatments. The genotypes are mostly clustered around the center of the plot. Those located on the right-side exhibit high performance such as Col 69, Col 9 and Col 96, with the best performing genotype in terms of yield, Col 93, positioned at the right edge for both treatments. Likewise, the genotype with the lowest yield Col 5 is found on the opposite side of the plot.

In the *ggplot* for AgNUE, genotypes positioned above the regression line represent those with superior performance under nitrogen low conditions (S1 Fig). The genotypes located in the upper right quadrant particularly Col 93, followed by Col 9 and Col 96 demonstrated high AgNUE in both environments, and can be considered efficient under contrasting nitrogen conditions. In contrast, genotypes such as Col 101, Col 115, and Col 91, located in the lower right quadrant, showed good performance under high nitrogen but poor efficiency under low nitrogen. Meanwhile, genotypes like Col 5, Galeras, and Col 21 show a low AgNUE under both nitrogen conditions (S1 Fig). Col 108 has a high percentage of nitrogen in tuber with AgNUE over the percentage of nitrogen in tuber under both conditions.

The results reveal a positive correlation between nitrogen and carbon accumulation in tubers, indicating that both nitrogen and carbon increase proportionally and are strongly associated with AgNUE (S2 Fig). Genotypes with higher nitrogen uptake and utilization accumulate more carbon, due to enhanced photosynthetic capacity and biomass production, where the C/N ratio agrees with the findings of [55], ranging from 10 to 50. Among all evaluated genotypes, Col 93 shows the highest AgNUE under both nitrogen conditions, nitrogen accumulation, and carbon accumulation, putting it as the most efficient genotype in this study. However, Col 93 also showed a marked decrease in the percentage of nitrogen allocated to tubers, dropping from 1.6% to 0.7% under high and low nitrogen conditions respectively, which negatively impacted tuber nutritional quality.

### Quantitative trait nucleotides (QTNs) identified for nitrogen use efficiency in the association panel

**Fig 3:**
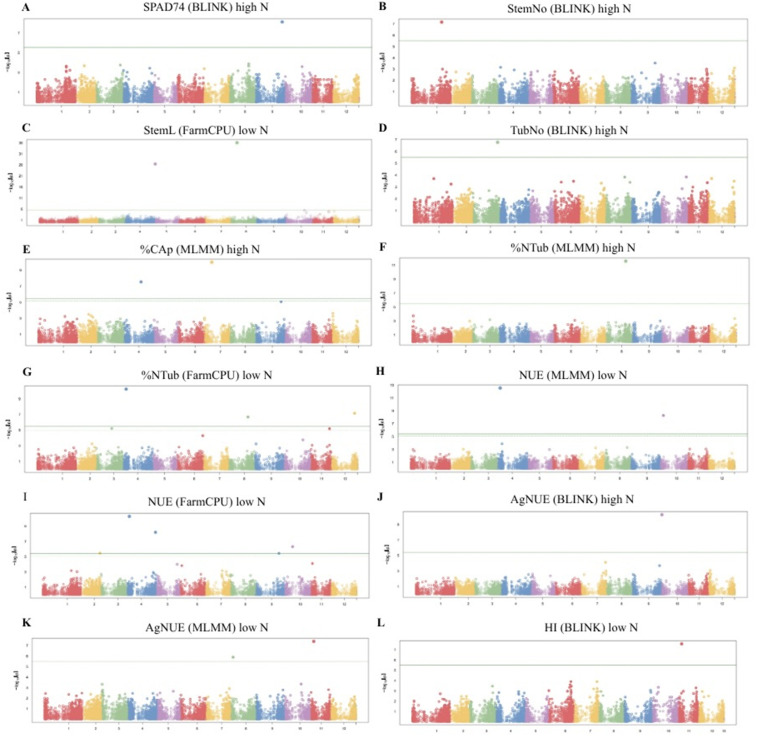
Manhattan plots displaying significant QTNs associated with NUE-related traits in diploid potato under high and low nitrogen conditions, analyzed with different statistical models. **A.** SPAD74 (BLINK) high nitrogen, **B.** StemNo (BLINK) high nitrogen, **C.** StemL (FarmCPU) low nitrogen, **D.** TubNo (BLINK) high nitrogen, **E.** %CAp (MLMM) high nitrogen, **F.** %Ntub (MLMM) high nitrogen, **G.** %Ntub (FarmCPU) low nitrogen, **H.** NUE (MLMM) low nitrogen, **I.** NUE (FarmCPU) low nitrogen, **J.** AgNUE (BLINK) high nitrogen, **K.** AgNUE (MLMM) low nitrogen, **L.** HI (BLINK) low nitrogen. The x axis represents the chromosomal position, while the y axis displays the − log₁₀ (p value) for QTNs associations. The horizontal green line indicates the genome wide significance threshold, with SNPs above this line considered statistically significant.

**Fig 4:**
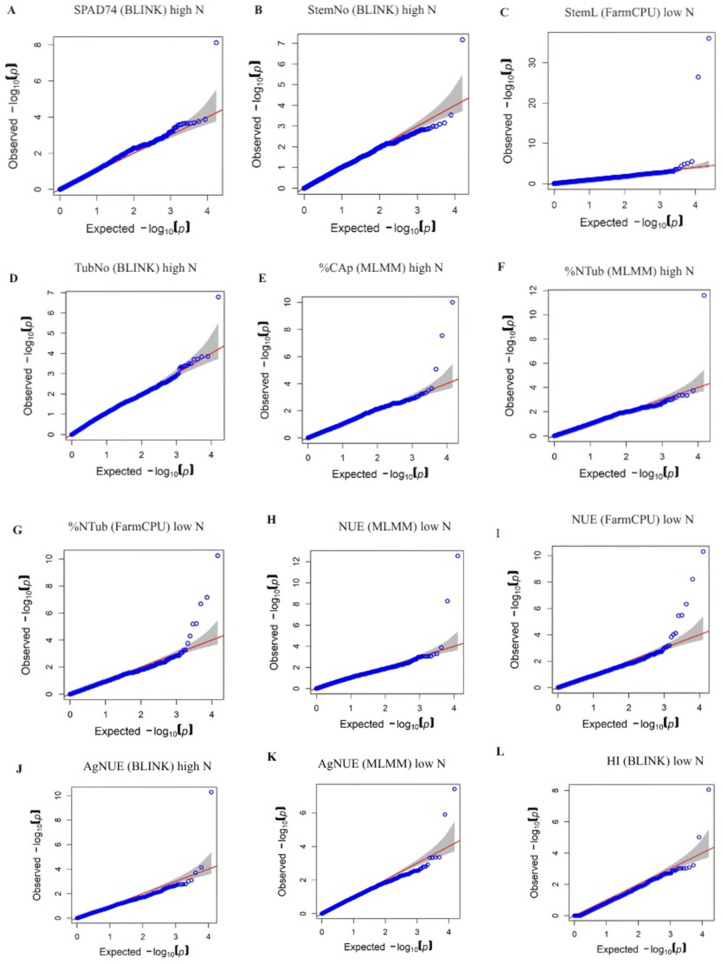
QQ plots showing the goodness of fit of the statistical models used for QTN identification associated with nitrogen use efficiency related traits in diploid potato under high and low nitrogen conditions. **A.** SPAD74 (BLINK) high nitrogen, **B**. StemNo (BLINK) high nitrogen, **C**. StemL (FarmCPU) low nitrogen, **D.** TubNo (BLINK) high nitrogen, **E**. %CAp (MLMM) high nitrogen, **F.** %Ntub (MLMM) high nitrogen, **G.** %Ntub (FarmCPU) low nitrogen, **H.** NUE (MLMM) low nitrogen, **I.** NUE (FarmCPU) low nitrogen, **J.** AgNUE (BLINK) high nitrogen, **K.** AgNUE (MLMM) low nitrogen, **L.** HI (BLINK) low nitrogen. The x axis represents the expected −log₁₀(*p*-value), while the y axis displays the observed −log₁₀(*p-*value) for variant trait associations.

The correction for potential population stratification effects applied to the GWAS models was defined using three principal components (PCs), which together explained 21.05% of the total variance (S3 Fig). Genome Wide Association Study identified a total of 21 QTNs significantly associated with 11 phenotypic variables related to nitrogen use efficiency (Table 1, Fig 3 and S6 Table). QQ plots confirmed a good fit of the association model applied to each phenotypic trait (Fig 4).

**Table 1:** Summary of QTNs and candidate genes in Linkage Disequilibrium (LD) for phenotypic variables and treatments of nitrogen use efficiency. Phenotypic variables, corresponding treatments of high and low nitrogen (N), total number of identified QTNs, and total number of candidate genes (GC) in linkage disequilibrium (LD) with each QTN.

Phenotypic variable	Total QTN	*p-*value range	p-value B&H correction	Total GC in LD
SPAD74 high N	1	7.50x10^-09^	1.30 x10^-04^	38
StemNo high N	1	6.70 x10^-08^	1x10^-03^	40
StemL low N	2	8.8 x10^-37^ to 3.50 x10^-27^	1.40 x10^-26^ to 4.20 x10^-19^	74
TubNo high N	1	1.6 x10^-07^	2.6 x10^-03^	53
%CAp high N	2	9.7 x10^-11^ to 2.80 x10^-08^	1.4 x10^-07^–2 x10^-04^	13
%Ntub high N	1	2.5 x10^-12^	3.6 x10^-08^	31
%Ntub low N	3	5.6 x10^-11^ to 2.07 x10^-07^	8.2 x10^-07^–1 x10^-03^	107
NUE low N	6	2.8 x10^-13^ to 3.6 x10^-06^	3.5 x10^-09^ to 9.23 x10^-03^	202
AgNUE high N	1	5.2 x10^-11^	6.4 x10^-07^	59
AgNUE low N	2	3.8 x10^-08^ to 1.20 x10^-06^	5.8 x10^-04^ to 9.50 x10^-03^	88
HI low N	1	2.8 x10^-08^	4.13 x10^-04^	45

The variables NUE low nitrogen and %Ntub low nitrogen exhibited the highest number of associated QTNs, with 6 and 3, respectively. The significance of the identified QTNs ranged from p-value of 8.8x10^-37^ to 3.6 x10^-6^, with QTN StemL low nitrogen located at Chromosome 8 position 15,178,651 showing the highest significance and QTN NUE low nitrogen located at Chromosome 8 position 54,180,237, the lowest. The phenotypic variance explained (PVE) by the identified QTNs ranged from 0.08% to 2.73%. The QTN associated with SPAD74 under high nitrogen, exhibited the lowest PVE (0.08%). In contrast, the QTN linked to stem length low nitrogen, showed the highest individual PVE (2.73%). Three additional QTNs related to NUE under low nitrogen conditions explained 1.62%, 1.99%, and 2.29% of the phenotypic variance. These variants together provide a high contribution to the genetic architecture of NUE (Table 1 and S6 Table).

### Repertoire of candidate genes identified for nitrogen use efficiency

From the 21 QTNs identified, nine localize with exonic regions, while 12 localize with intronic regions. Among those located in coding regions, the analysis revealed that all QTNs caused nonsynonymous changes in the gene’s codon, potentially altering protein function (S6 Table). The analysis of LD decay revealed a drop at 263 Kb (S4 Fig). Consequently, this window size around the identified QTNs was used for the search of candidate genes (CGs) in linkage disequilibrium with the associated variable. The total number of CGs identified for the 11 phenotypic variables for which QTNs were detected was 750. The number of CGs ranged from 13 for the %CAp high nitrogen variable to 202 for NUE under low nitrogen conditions (Table 1 and S7 Table).

Within the repertoire of candidate genes, several genes were found whose functional annotation are related to specialized roles in plant nitrogen metabolism. These include genes involved in multiple physiological and regulatory pathways associated with NUE, that can be classified in *nitrogen uptake nitrogen utilization, nitrogen transport and NUE hormonal regulation*. Candidate genes in the *nitrogen uptake category* stand out, such as genes encoding nutrient transporters, including those for nitrogen: (Soltu.DM.12G023520.1, Soltu.DM.05G000760.3), potassium (Soltu.DM.02G032740.1, Soltu.DM.10G001150.1), magnesium (Soltu.DM.01G027980.1, Soltu.DM.05G000840.1), sulfur (Soltu.DM.09G024940.1), zinc (Soltu.DM.10G006310.1), iron (Soltu.DM.09G025190.1), and copper (Soltu.DM.10G000590.1). Also, a *MYB* family member (Soltu.DM.02G033070.1) a gene coding for a phosphate deficiency response (PHR) transcription factor. (S7 Table). Within the *nitrogen utilization* were found CGs coding for an Isocitrate Dehydrogenase (IDH) (Soltu.DM.02G033150.1), an enzyme Ornithine carbamoyltransferase (Soltu.DM.04G035700.1), a gene that encodes a Patatin like protein (Soltu.DM.09G019820.1), and two genes coding for proteins involved in protein degradation (Soltu.DM.11G007670.3) and (Soltu.DM.04G036070.1).

In the *nitrogen transport* category, the gene Soltu.DM.05G000760.3, which encodes a ureide permease, was identified, along with several members of the ATP binding cassette (ABC) transporter family (Soltu.DM.04G007120.1 and Soltu.DM.04G007130.1). Regarding hormonal regulation related to NUE, the candidate gene set included an amidase family protein (Soltu.DM.03G032260.2) and a gene encoding an Indole-3-butyric acid (IBA) response protein (Soltu.DM.12G023410.1). Additionally, genes associated with root hair development and ethylene signaling were identified, such as *Cysteine_synthase_C1* (Soltu.DM.10G006300.1), along with two genes involved in the cytokinin biosynthetic pathway: tRNA isopentenyltransferase (Soltu.DM.10G006560.1 and Soltu.DM.10G006610.1), both encoding isopentenyltransferases. Finally, a gene related to carbon sequestration was identified: Chlorophyll AB binding protein (Soltu.DM.09G025070.1) (S4 Table).

## Discussion

Here we are reporting a total number of CGs identified for NUE for the 11 phenotypic variables measured for which QTNs were detected was 750, which contributes to deciphering the genetic structure underlying NUE in *Solanum tuberosum*, one of the world’s most important crops. This is a fundamental step for understanding the genetic control of this trait and a basic contribution to the plant breeding knowledge toward more sustainable and environmentally friendly agriculture. Nitrogen is an essential nutrient for plant growth and productivity and is used in large quantities in agriculture [56]. However, its excessive and inefficient use in crops like potato raises environmental concerns due to greenhouse gas emissions, water pollution, and disruption of nearby ecosystems [22,57]. Understanding the genetic regulation of NUE and applying this knowledge to potato breeding could reduce fertilizer use, increase production efficiency, and mitigate environmental impacts.

The candidate gene repertoire identified through GWAS in this study reveals a wide array of genomic regions potentially involved in regulating NUE in potato. While future functional validation of these CGs is needed. The current functional annotation of these CGs offers valuable clues to guide such efforts. We focused on four functional categories related to nitrogen metabolism: *nitrogen uptake, nitrogen utilization, nitrogen transport, and hormonal regulation of NUE.*

### Nitrogen uptake

Membrane transporters are key regulators of nitrogen and other essential nutrient uptake and distribution throughout the plant [58]. We identified genes encoding transporters for nitrogen, potassium, magnesium, sulfur, zinc, iron, and cooper. These were associated with nearly all evaluated traits, suggesting NUE in potato depends on coordinated regulation of multiple nutrient transport systems, not only nitrogen specific ones. This aligns with recent evidence on nutrient crosstalk, showing that nitrogen availability can modulate the expression of phosphate deficiency response (PHR) transcription factors members of the MYB family [59], such as Soltu.DM.02G033070.1 identified in this study. PHRs regulate phosphorus transporter expression and have been proposed as master regulators integrating nitrogen, phosphorus, sulfur, and iron homeostasis [60], supporting the idea that NUE relies on integrated nutrient uptake regulation. One key CG identified was Soltu.DM.12G023520.1, encoding a nitrate transporter of the NRT1/PTR family [61]. NRT1s are low affinity transporters essential for nitrate uptake and movement across membranes. In rice, overexpression of NRT1 members improves NUE and biomass production [62–64]. These transporters facilitate cell to cell nitrate movement and enhance soil nitrate uptake, crucial for nitrogen utilization.

### Nitrogen utilization

In the nitrogen assimilation pathway, ammonium is transferred to 2-oxoglutarate to produce glutamate, a key amino acid precursor [65]. The *IDH* gene (Soltu.DM.02G033150.1), associated with NUE under low nitrogen in our study, is involved in 2-oxoglutarate synthesis [66], suggesting it could be a rate limiting enzyme in this pathway. Another gene, Soltu.DM.04G035700.1, encodes ornithine carbamoyltransferase, which uses glutamate to synthesize arginine, a nitrogen storage compound and nitric oxide (NO) precursor, essential for root development [67]. We also identified Soltu.DM.09G019820.1, encoding a Patatin like protein that represents over 45% of tuber nitrogen and is known to increase concentration with higher soil nitrogen [68]. Additionally, we found genes related to protein degradation, a process critical for Nitrogen recycling from leaves. In *Arabidopsis*, overexpression of UBP12 and UBP13 (Soltu.DM.11G007670.3) accelerates senescence under nitrogen deficiency, facilitating nitrogen remobilization to growing tissues [69]. A similar role is played by SAG12 (Soltu.DM.04G036070.1), a papain-like cysteine protease, involved in protein degradation during senescence [70].

Col 93 showed a marked decrease in the percentage of nitrogen allocated to tubers, dropping from 1,6% to 0,7% under high and low nitrogen conditions respectively, which negatively impacted tuber nutritional quality. This reduction suggests that most nitrogen corresponds to non-essential storage nitrogen forms, rather than nitrogen required by the growing tuber tissues [68].

### Nitrogen transport

Allantoin, a ureide derived from nucleic acid degradation, is involved in nitrogen recycling [71]. In potato, 10 members of the UPS family have been identified, including the allantoin, they play an important role in nitrogen transport [72]. In *Arabidopsis*, ureides are transported from senescing to developing tissues by ureide permeases [73]. We identified a homologous gene (Soltu.DM.05G000760.3), suggesting a role in long distance nitrogen transport. Proteins are a major nitrogen source in senescing leaves, contributing over 50% to nitrogen remobilization [71]. Also, we identified the genes Soltu.DM.04G007120.1 and Soltu.DM.04G007130.1, which are involved in the export of metabolites, mostly amino acids, peptides, or small proteins. This transport is facilitated by protein transporters such as ATP-Binding Cassette (ABC) transporters [74]. These encode H⁺ pumps that drive molecule transport using electrochemical gradients [75]. In Arabidopsis, ABC transporters are also crucial for auxin transport, influencing root growth [76]. The identification of these transporters as nitrate transporters in cyanobacteria [77], provides a basis to investigate their potential role in nitrate uptake and transport in potato.

### Hormonal regulation of nitrogen use efficiency

Hormones like auxins and ethylene play key roles in root development, especially under limited nutrient conditions, by improving soil exploration and nutrient uptake. Several CGs involved in hormonal signaling were identified, including an amidase (Soltu.DM.03G032260.2) that catalyzes IAM to IAA an active auxin [78], and an indole-3-butyric acid (IBA) response protein (Soltu.DM.12G023410.1), promoter of root growth in potato [79]. In *Arabidopsis*, overexpression of AMI1 (amidase family) significantly increases the number of adventitious roots [78,80]. We also found CGs involved in ethylene signaling and in the regulation of root hair development. Cysteine_synthase_C1 (Soltu.DM.10G006300.1) was associated with NUE under low nitrogen. In *Arabidopsis*, knockout of this gene impairs root development without affecting shoots It also upregulates ethylene related genes like arabinogalactan protein (Soltu.DM.08G004630.1) and ACC synthase (Soltu.DM.08G004500.1) [81]. ACC, an ethylene precursor, affects root architecture, especially lateral root elongation [82,83].

We also found genes in the cytokinin biosynthetic pathway, including tRNA isopentenyltransferase (Soltu.DM.10G006560.1, Soltu.DM.10G006610.1) and a gene cluster (Soltu.DM.10G006390.1 to Soltu.DM.10G006620.1) encoding isopentenyltransferase key enzymes for cytokinin biosynthesis [84]. Cytokinins regulate shoot meristem activity and suppress root meristem activity [85]. In rice, low nitrogen reduces cytokinin levels, promoting seminal root growth via meristem cell proliferation and elongation [86]. Multiple gene copies suggest a duplication event that could enhance cytokinin production and influence the lateral root development under limited nitrogen conditions [87].

In this study, ammonium nitrate was used as a nitrogen source, beyond its nutritional role, nitrate is also recognized as a signaling molecule that induces cytokinin biosynthesis in roots, as extensively reported in the literature [88] and the presence of multiple related genes may reflect a enhances cytokinin production, potentially influencing root development.

A gene associated with carbon accumulation as well as NUE, was also identified: high chlorophyll fluorescence and chlorophyll a/b binding protein (Soltu.DM.04G017810.2 and Soltu.DM.09G025070.1, respectively), both linked to photosystems I and II. These proteins capture and transfer excitation energy under different light intensities and help balance energy distribution, thereby increasing light use efficiency and potentially enhancing carbon assimilation in leaves [17,89].

Given NUE has strong environmental dependency, accurate phenotyping is essential [90]. We applied a substrate grow system under low nitrogen conditions to precisely assess NUE related traits at key growth stages. This method can serve as a reference for future research in crops with similar agronomic traits. Incorporating high throughput phenotyping, especially in the field, would further refine physiological measurements. Finally, the QTNs and CGs identified through GWAS offer promising targets for potato breeding. Prioritization and validation of top CGs via gene editing or overexpression could clarify their roles in NUE and support the development of potato varieties with lower fertilizer needs and contribute to sustainable agriculture. In this context, functional validation of the candidate genes could be achieved through advanced molecular approaches such as gene editing and genetic transformation. Techniques like CRISPR/Cas9 [91] offer the possibility to generate targeted knockouts or allelic variants to test the role of specific genes in nitrogen uptake and utilization. Likewise, stable or transient transformation systems in potato and model plants could be used to assess the expression patterns and phenotypic effects of these candidate genes. Such approaches would not only provide direct evidence for their involvement in NUE but also accelerate the translation of genomic discoveries into practical applications, facilitating the development of cultivars with improved nitrogen efficiency.

The extrapolation of these findings from diploid potatoes to commercial tetraploid cultivars may represent a challenge. However, Solanum tuberosum Group Phureja is known for producing unreduced (2n) gametes [92–94], which enables their use in crosses with tetraploid cultivars and facilitates the introgression of desirable traits through backcrossing [95].

The genotypes Col 5, Galeras, and Col 21 exhibited low nitrogen use efficiency under both high and low nitrogen conditions. One possible explanation is that these accessions were not selected for yield related traits but rather preserved due to their genetic diversity. Consequently, they do not appear to carry favorable alleles associated with efficient nitrogen assimilation and remobilization. In our GWAS analysis, we identified several candidate genes involved in nitrate transport (NRT family), carbon-nitrogen transport, and root development, which showed weak or no association with these genotypes. This could partially explain their low performance under both nitrogen regimes.

## Conclusion

The QTNs and candidate genes identified represent a major step forward in further dissecting the NUE in potato. Nevertheless, the polygenic nature of the trait implies that it is influenced by the interaction of multiple genes whose roles are determined by different physiological mechanisms. Thus, the findings described here require further research and functional validation efforts to contribute to our understanding of the underlying biological process but also to allow, in the near future, the development of cultivars with optimized nitrogen yield, reducing the need for synthetic fertilizers, which is especially important in the global context of rising food demand and restrictions on the use of agricultural inputs.

## Supporting information

S1 Table Association panel. (XLSX)

S2 Table SNPs matrix. (TXT)

S3 Table ANOVA of the variables evaluated under high and low N conditions. (DOCX)

S4 Table PCA scores, loadings, eigenvalues, individual contributions, and variable contributions under low N conditions. (XLSX)

S5 Table PCA scores, loadings, eigenvalues, individual contributions, and variable contributions under high N conditions. (XLSX)

S6 Table List of QTNs identified by GWAS models. (XLSX)

S7 Table Repertoire of candidate genes. (XLSX)

S1 Fig Relationship between Agronomic Nitrogen Use Efficiency (AgNUE) under high and low nitrogen conditions. Each point represents a genotype. The color gradient indicates the percentage of nitrogen accumulated in tubers under low nitrogen conditions (%NtubLowN), with green tones indicating lower values and darker tones higher values.
(TIFF)

S2 Fig Relationship between accumulated carbon and nitrogen in tubers under low nitrogen conditions. Each point represents a genotype. The color gradient indicates Agronomic Nitrogen Use Efficiency (AgNUE), with green tones representing lower values and darker tones higher values.
(TIFF)

S3 Fig PCA for population structure. (TIFF)

S4 Fig Linkage disequilibrium (LD) decay. (TIFF)

## References

[1] Koch M, Naumann M, Pawelzik E, Gransee A, Thiel H. The importance of nutrient management for potato production part I: plant nutrition and yield. Potato Res. 2019;63(1):97–119. doi: 10.1007/s11540-019-09431-2

[2] Hu C, He Y, Zhang W, He J. Potato proteins for technical applications: Nutrition, isolation, modification and functional properties - A review. Innov Food Sci Emerg Tech. 2024;91:103533. doi: 10.1016/j.ifset.2023.103533

[3] Narváez-Cuenca C-E, Peña C, Restrepo-Sánchez L-P, Kushalappa A, Mosquera T. Macronutrient contents of potato genotype collections in the *Solanum tuberosum* Group Phureja. J Food Comp Anal. 2018;66:179–84. doi: 10.1016/j.jfca.2017.12.019

[4] Parra-Galindo M-A, Piñeros-Niño C, Soto-Sedano JC, Mosquera-Vasquez T. Chromosomes I and X harbor consistent genetic factors associated with the anthocyanin variation in potato. Agronomy. 2019;9(7):366. doi: 10.3390/agronomy9070366

[5] Riveros-Loaiza LM, Benhur-Cardona N, Lopez-Kleine L, Soto-Sedano JC, Pinzón AM, Mosquera-Vásquez T, et al. Uncovering anthocyanin diversity in potato landraces (Solanum tuberosum L. Phureja) using RNA-seq. PLoS One. 2022;17(9):e0273982. doi: 10.1371/journal.pone.0273982 36136976 PMC9498938

[6] Su D, Zhang H, Teng A, Zhang C, Lei L, Ba Y, et al. Potato growth, nitrogen balance, quality, and productivity response to water-nitrogen regulation in a cold and arid environment. Front Plant Sci. 2024;15:1451350. doi: 10.3389/fpls.2024.1451350 39479537 PMC11521918

[7] Pimentel D. Green revolution agriculture and chemical hazards. Sci Total Environ. 1996;188 Suppl 1:S86-98. doi: 10.1016/0048-9697(96)05280-1 8966546

[8] Näsholm T, Kielland K, Ganeteg U. Uptake of organic nitrogen by plants. New Phytol. 2009;182(1):31–48. doi: 10.1111/j.1469-8137.2008.02751.x 19210725

[9] Khush GS. Green revolution: preparing for the 21st century. Genome. 1999;42(4):646–55. doi: 10.1139/g99-044 10464789

[10] Shen J, Li Y, Liu X, Luo X, Tang H, Zhang Y, et al. Atmospheric dry and wet nitrogen deposition on three contrasting land use types of an agricultural catchment in subtropical central China. Atmosp Environ. 2013;67:415–24. doi: 10.1016/j.atmosenv.2012.10.068

[11] Hirel B, Krapp A. Amino Acids | Nitrogen utilization in plants I biological and agronomic importance. In: Encyclopedia of biological chemistry III. Elsevier; 2021. 127–40. doi: 10.1016/b978-0-12-809633-8.21265-x

[12] Li Q, Lu X, Wang C, Shen L, Dai L, He J, et al. Genome-wide association study and transcriptome analysis reveal new QTL and candidate genes for nitrogen‐deficiency tolerance in rice. Crop J. 2022;10(4):942–51. doi: 10.1016/j.cj.2021.12.006

[13] Getahun BB, Tiruneh MA, Aliche E, Malossetti M, Visser RG, van der Linden CG. Genotype-by-environment interaction for quantitative trait loci affecting nitrogen use efficiency and associated traits in potato. Potato Res. 2022;65(4):777–807. doi: 10.1007/s11540-022-09548-x

[14] Shrestha V, Chhetri HB, Kainer D, Xu Y, Hamilton L, Piasecki C, et al. The genetic architecture of nitrogen use efficiency in switchgrass (Panicum virgatum L.). Front Plant Sci. 2022;13:893610. doi: 10.3389/fpls.2022.893610 35586220 PMC9108870

[15] Siddiqui MN, Pandey K, Bhadhury SK, Sadeqi B, Schneider M, Sanchez-Garcia M, et al. Convergently selected NPF2.12 coordinates root growth and nitrogen use efficiency in wheat and barley. New Phytol. 2023;238(5):2175–93. doi: 10.1111/nph.18820 36808608

[16] Yu K, Liu J, Sun M, Ma X, Han B, Li M, et al. Candidate gene discovery for nitrogen use efficiency in rice based on genome-wide association study. Curr Plant Biol. 2025;42:100479. doi: 10.1016/j.cpb.2025.100479

[17] Zhang Z, Peng C, Xu W, Li Y, Qi X, Zhao M. Genome-wide association study of agronomic traits related to nitrogen use efficiency in Henan wheat. BMC Genomics. 2024;25(1):7. doi: 10.1186/s12864-023-09922-0 38166525 PMC10759698

[18] Hirschhorn JN, Daly MJ. Genome-wide association studies for common diseases and complex traits. Nat Rev Genet. 2005;6(2):95–108. doi: 10.1038/nrg1521 15716906

[19] Sood S, Bhardwaj V, Mangal V, Kumar A, Singh B, Dipta B, et al. Genome-wide association mapping to identify genetic loci governing agronomic traits and genomic prediction prospects in tetraploid potatoes. Sci Hortic. 2024;328:112900. doi: 10.1016/j.scienta.2024.112900

[20] Wang Y, Zhu T, Yang J, Wang H, Ji W, Xu Y, et al. GWAS and transcriptome analysis reveal key genes affecting root growth under low nitrogen supply in maize. Genes (Basel). 2022;13(9):1632. doi: 10.3390/genes13091632 36140800 PMC9498817

[21] Fan X, Tang Z, Tan Y, Zhang Y, Luo B, Yang M, et al. Overexpression of a pH-sensitive nitrate transporter in rice increases crop yields. Proc Natl Acad Sci U S A. 2016;113(26):7118–23. doi: 10.1073/pnas.1525184113 27274069 PMC4932942

[22] Getahun BB, Visser RGF, van der Linden CG. Identification of QTLs associated with nitrogen use efficiency and related traits in a diploid potato population. Am J Potato Res. 2020;97(2):185–201. doi: 10.1007/s12230-020-09766-4

[23] Iribar C, Alvarez-Morezuelas A, Barandalla L, Ruiz de Galarreta JI. Genome-wide association analysis of traits related to nitrogen deficiency stress in potato. Horticulturae. 2025;11(8):889. doi: 10.3390/horticulturae11080889

[24] Ospina Nieto CA, Lammerts van Bueren ET, Allefs S, Vos PG, van der Linden G, Maliepaard CA, et al. Association mapping of physiological and morphological traits related to crop development under contrasting nitrogen inputs in a diverse set of potato cultivars. Plants (Basel). 2021;10(8):1727. doi: 10.3390/plants10081727 34451774 PMC8398069

[25] Geng Y, Baumann F, Song C, Zhang M, Shi Y, Kühn P, et al. Increasing temperature reduces the coupling between available nitrogen and phosphorus in soils of Chinese grasslands. Sci Rep. 2017;7:43524. doi: 10.1038/srep43524 28266635 PMC5339893

[26] Ma J-Y, Sun W, Liu X-N, Chen F-H. Variation in the stable carbon and nitrogen isotope composition of plants and soil along a precipitation gradient in northern China. PLoS One. 2012;7(12):e51894. doi: 10.1371/journal.pone.0051894 23272186 PMC3525597

[27] Juyó D, Sarmiento F, Álvarez M, Brochero H, Gebhardt C, Mosquera T. Genetic diversity and population structure in diploid potatoes of Solanum tuberosum Group Phureja. Crop Science. 2015;55(2):760–9. doi: 10.2135/cropsci2014.07.0524

[28] Parra-Galindo MA, Soto-Sedano JC, Mosquera-Vásquez T, Roda F. Pathway-based analysis of anthocyanin diversity in diploid potato. PLoS One. 2021;16(4):e0250861. doi: 10.1371/journal.pone.0250861 33914830 PMC8084248

[29] Ghislain M, Andrade D, Rodríguez F, Hijmans RJ, Spooner DM. Genetic analysis of the cultivated potato Solanum tuberosum L. Phureja Group using RAPDs and nuclear SSRs. Theor Appl Genet. 2006;113(8):1515–27. doi: 10.1007/s00122-006-0399-7 16972060

[30] Diaz-Valencia P, Melgarejo LM, Arcila I, Mosquera-Vásquez T. Physiological, biochemical and yield-component responses of Solanum tuberosum L. group phureja genotypes to a water deficit. Plants (Basel). 2021;10(4):638. doi: 10.3390/plants10040638 33801743 PMC8065493

[31] Potato Genome Sequencing Consortium, Xu X, Pan S, Cheng S, Zhang B, Mu D, et al. Genome sequence and analysis of the tuber crop potato. Nature. 2011;475(7355):189–95. doi: 10.1038/nature10158 21743474

[32] Jiménez-Medrano A, Mendoza-Bustamante M, Rueda-Carvajal N, Magnitskiy S, Mosquera-Vásquez T. Nitrogen use efficiency in diploid potato of the Phureja group: a novel insight. Potato Res. 2025. doi: 10.1007/s11540-025-09932-3

[33] Arnold CY. The determination and significance of the base temperature in a linear heat unit system. HortScience. 1959;74:430–45. doi: 10.5555/19600303187

[34] Nguyen GN, Kant S. Improving nitrogen use efficiency in plants: effective phenotyping in conjunction with agronomic and genetic approaches. Funct Plant Biol. 2018;45(6):606–19. doi: 10.1071/FP17266 32290963

[35] Liu K, Seegers S, Hojilla‐Evangelista MP, Pallares Pallares A, Wu X. International collaborative study on measuring protein solubility index for legumes, oilseeds, cereals, and related products. Sustain Food Proteins. 2024;2(4):236–49. doi: 10.1002/sfp2.1039

[36] Rhezali A, Aissaoui AE. Feasibility study of using absolute SPAD values for standardized evaluation of corn nitrogen status. Nitrogen. 2021;2(3):298–307. doi: 10.3390/nitrogen2030020

[37] Zebarth BJ, Tarn TR, de Jong H, Murphy A. Nitrogen use efficiency characteristics of andigena and diploid potato selections. Am J Pot Res. 2008;85(3):210–8. doi: 10.1007/s12230-008-9014-6

[38] Tiwari JK, Devi S, Buckseth T, Ali N, Singh RK, Zinta R, et al. Precision phenotyping of contrasting potato (Solanum tuberosum L.) varieties in a novel aeroponics system for improving nitrogen use efficiency: in search of key traits and genes. J Integ Agric. 2020;19(1):51–61. doi: 10.1016/s2095-3119(19)62625-0

[39] Lê S, Josse J, Husson F. FactoMineR: an R package for multivariate analysis. J Stat Softw. 2008;25(1):1–18.

[40] Kassambara A, Mundt F. Factoextra: Extract and Visualize the Results of Multivariate Data Analyses. 2016. https://cran.r-project.org/web/packages/factoextra/readme/README.html

[41] Villanueva RAM, Chen ZJ. ggplot2: Elegant Graphics for Data Analysis (2nd ed.). Measurement: Interdisciplinary Research and Perspectives. 2019;17(3):160–7. doi: 10.1080/15366367.2019.1565254

[42] Juyo Rojas DK, Soto Sedano JC, Ballvora A, Léon J, Mosquera Vásquez T. Novel organ-specific genetic factors for quantitative resistance to late blight in potato. PLoS One. 2019;14(7):e0213818. doi: 10.1371/journal.pone.0213818 31310605 PMC6634379

[43] Pham GM, Hamilton JP, Wood JC, Burke JT, Zhao H, Vaillancourt B, et al. Construction of a chromosome-scale long-read reference genome assembly for potato. Gigascience. 2020;9(9):giaa100. doi: 10.1093/gigascience/giaa100 32964225 PMC7509475

[44] Brown J, Pirrung M, McCue LA. FQC dashboard: integrates FastQC results into a web-based, interactive, and extensible FASTQ quality control tool. Bioinformatics. 2017;33(19):3137–9. doi: 10.1093/bioinformatics/btx373 28605449 PMC5870778

[45] Duitama J, Quintero JC, Cruz DF, Quintero C, Hubmann G, Foulquié-Moreno MR, et al. An integrated framework for discovery and genotyping of genomic variants from high-throughput sequencing experiments. Nucleic Acids Res. 2014;42(6):e44. doi: 10.1093/nar/gkt1381 24413664 PMC3973327

[46] Bradbury PJ, Zhang Z, Kroon DE, Casstevens TM, Ramdoss Y, Buckler ES. TASSEL: software for association mapping of complex traits in diverse samples. Bioinformatics. 2007;23(19):2633–5. doi: 10.1093/bioinformatics/btm308 17586829

[47] Huang M, Liu X, Zhou Y, Summers RM, Zhang Z. BLINK: a package for the next level of genome-wide association studies with both individuals and markers in the millions. Gigascience. 2019;8(2):giy154. doi: 10.1093/gigascience/giy154 30535326 PMC6365300

[48] Segura V, Vilhjálmsson BJ, Platt A, Korte A, Seren Ü, Long Q, et al. An efficient multi-locus mixed-model approach for genome-wide association studies in structured populations. Nat Genet. 2012;44(7):825–30. doi: 10.1038/ng.2314 22706313 PMC3386481

[49] Liu X, Huang M, Fan B, Buckler ES, Zhang Z. Iterative Usage of Fixed and Random Effect Models for Powerful and Efficient Genome-Wide Association Studies. PLoS Genet. 2016;12(2):e1005767. doi: 10.1371/journal.pgen.1005767 26828793 PMC4734661

[50] Lipka AE, Tian F, Wang Q, Peiffer J, Li M, Bradbury PJ, et al. GAPIT: genome association and prediction integrated tool. Bioinformatics. 2012;28(18):2397–9. doi: 10.1093/bioinformatics/bts444 22796960

[51] Benjamini Y, Hochberg Y. Controlling the false discovery rate: a practical and powerful approach to multiple testing. J Royal Stat Soc Series B: Stat Methodol. 1995;57(1):289–300. doi: 10.1111/j.2517-6161.1995.tb02031.x

[52] Lamesch P, Berardini TZ, Li D, Swarbreck D, Wilks C, Sasidharan R, et al. The Arabidopsis Information Resource (TAIR): improved gene annotation and new tools. Nucleic Acids Res. 2012;40(Database issue):D1202-10. doi: 10.1093/nar/gkr1090 22140109 PMC3245047

[53] El-Gebali S, Mistry J, Bateman A, Eddy SR, Luciani A, Potter SC, et al. The Pfam protein families database in 2019. Nucleic Acids Res. 2019;47(D1):D427–32. doi: 10.1093/nar/gky995 30357350 PMC6324024

[54] Wu CH, Apweiler R, Bairoch A, Natale DA, Barker WC, Boeckmann B, et al. The Universal Protein Resource (UniProt): an expanding universe of protein information. Nucleic Acids Res. 2006;34(Database issue):D187-91. doi: 10.1093/nar/gkj161 16381842 PMC1347523

[55] Zheng H, Wang Y, Zhao J, Shi X, Ma Z, Fan M. Tuber formation as influenced by the C : N ratio in potato plants. J Plant Nutr Soil Sci. 2018;181(5):686–93. doi: 10.1002/jpln.201700571

[56] Hirel B, Krapp A. Amino Acids | Nitrogen utilization in plants I biological and agronomic importance. Encyclopedia of biological chemistry III. Elsevier; 2021. 127–40. doi: 10.1016/b978-0-12-809633-8.21265-x

[57] Saravia D, Farfán-Vignolo ER, Gutiérrez R, De Mendiburu F, Schafleitner R, Bonierbale M, et al. Yield and physiological response of potatoes indicate different strategies to cope with drought stress and nitrogen fertilization. Am J Potato Res. 2016;93(3):288–95. doi: 10.1007/s12230-016-9505-9

[58] Sharma R, Kumar D, Parkirti P, Singh A, Sharma A, Langeh K, et al. Membrane transporters in Plants: key players in abiotic and biotic stress tolerance and nutritional transport. Plant Physiol Biochem. 2025;227:110084. doi: 10.1016/j.plaphy.2025.110084 40449185

[59] Fan X, Zhou X, Chen H, Tang M, Xie X. Cross-talks between macro- and micronutrient uptake and signaling in plants. Front Plant Sci. 2021;12:663477. doi: 10.3389/fpls.2021.663477 34721446 PMC8555580

[60] Kumar S, Kumar S, Mohapatra T. Interaction between macro- and micro-nutrients in plants. Front Plant Sci. 2021;12:665583. doi: 10.3389/fpls.2021.665583 34040623 PMC8141648

[61] Nedelyaeva OI, Khramov DE, Balnokin YV, Volkov VS. Functional and molecular characterization of plant nitrate transporters belonging to NPF (NRT1/PTR) 6 subfamily. Int J Mol Sci. 2024;25(24):13648. doi: 10.3390/ijms252413648 39769409 PMC11677463

[62] Fang Z, Xia K, Yang X, Grotemeyer MS, Meier S, Rentsch D, et al. Altered expression of the PTR/NRT1 homologue OsPTR9 affects nitrogen utilization efficiency, growth and grain yield in rice. Plant Biotechnol J. 2013;11(4):446–58. doi: 10.1111/pbi.12031 23231455

[63] Wang W, Hu B, Yuan D, Liu Y, Che R, Hu Y, et al. Expression of the nitrate transporter gene OsNRT1.1A/OsNPF6.3 confers high yield and early maturation in rice. Plant Cell. 2018;30(3):638–51. doi: 10.1105/tpc.17.00809 29475937 PMC5894839

[64] Yang X, Nong B, Chen C, Wang J, Xia X, Zhang Z, et al. OsNPF3.1, a member of the NRT1/PTR family, increases nitrogen use efficiency and biomass production in rice. The Crop Journal. 2023;11(1):108–18. doi: 10.1016/j.cj.2022.07.001

[65] Forde BG, Lea PJ. Glutamate in plants: metabolism, regulation, and signalling. J Exp Bot. 2007;58(9):2339–58. doi: 10.1093/jxb/erm121 17578865

[66] Wei N, Zhang Z, Yang H, Hu D, Wu Y, Xue J, et al. Characterization of the isocitrate dehydrogenase gene family and their response to drought stress in maize. Plants (Basel). 2023;12(19):3466. doi: 10.3390/plants12193466 37836206 PMC10574653

[67] Urbano-Gámez JA, El-Azaz J, Ávila C, de la Torre FN, Cánovas FM. Enzymes involved in the biosynthesis of arginine from ornithine in maritime pine (Pinus pinaster Ait.). Plants (Basel). 2020;9(10):1271. doi: 10.3390/plants9101271 32992504 PMC7601404

[68] Dimante I, Skrabule I, Sokolova E, Taskova I, Berga D, Sterna V. Exploring the relationship between nitrogen use efficiency and protein concentrations in potato genotypes. Agronomy. 2024;14(7):1517. doi: 10.3390/agronomy14071517

[69] Park S-H, Jeong JS, Seo JS, Park BS, Chua N-H. Arabidopsis ubiquitin-specific proteases UBP12 and UBP13 shape ORE1 levels during leaf senescence induced by nitrogen deficiency. New Phytol. 2019;223(3):1447–60. doi: 10.1111/nph.15879 31050353

[70] Liu H, Hu M, Wang Q, Cheng L, Zhang Z. Role of papain-like cysteine proteases in plant development. Front Plant Sci. 2018;9:1717. doi: 10.3389/fpls.2018.01717 30564252 PMC6288466

[71] Soltabayeva A, Srivastava S, Kurmanbayeva A, Bekturova A, Fluhr R, Sagi M. Early senescence in older leaves of low nitrate-grown Atxdh1 uncovers a role for purine catabolism in N supply. Plant Physiol. 2018;178(3):1027–44. doi: 10.1104/pp.18.00795 30190419 PMC6236613

[72] Huang W, Lu Y, Ren B, Zeng F, Liu Y, Lu L, et al. Identification and expression analysis of UPS gene family in potato. Genes (Basel). 2024;15(7):870. doi: 10.3390/genes15070870 39062649 PMC11275393

[73] Lescano I, Bogino MF, Martini C, Tessi TM, González CA, Schumacher K, et al. Ureide permease 5 (AtUPS5) connects cell compartments involved in ureide metabolism. Plant Physiol. 2020;182(3):1310–25. doi: 10.1104/pp.19.01136 31862838 PMC7054880

[74] Dahuja A, Kumar RR, Sakhare A, Watts A, Singh B, Goswami S, et al. Role of ATP-binding cassette transporters in maintaining plant homeostasis under abiotic and biotic stresses. Physiol Plant. 2021;171(4):785–801. doi: 10.1111/ppl.13302 33280130

[75] Rea PA. Plant ATP-binding cassette transporters. Annu Rev Plant Biol. 2007;58:347–75. doi: 10.1146/annurev.arplant.57.032905.105406 17263663

[76] Jenness MK, Tayengwa R, Bate GA, Tapken W, Zhang Y, Pang C, et al. Loss of multiple ABCB auxin transporters recapitulates the major twisted dwarf 1 phenotypes in Arabidopsis thaliana. Front Plant Sci. 2022;13:840260. doi: 10.3389/fpls.2022.840260 35528937 PMC9069160

[77] Li B, Wang X-Q, Li Q-Y, Xu D, Li J, Hou W-T, et al. Allosteric regulation of nitrate transporter NRT via the signaling protein PII. Proc Natl Acad Sci U S A. 2024;121(11):e2318320121. doi: 10.1073/pnas.2318320121 38457518 PMC10945777

[78] Pérez-Alonso M-M, Ortiz-García P, Moya-Cuevas J, Lehmann T, Sánchez-Parra B, Björk RG, et al. Endogenous indole-3-acetamide levels contribute to the crosstalk between auxin and abscisic acid, and trigger plant stress responses in Arabidopsis. J Exp Bot. 2021;72(2):459–75. doi: 10.1093/jxb/eraa485 33068437 PMC7853601

[79] Kumari M. Effect of indole-3 butyric acid application on micro plants of potato (Solanum tuberosum L.). Agricultural Research Journal. 2021;58(6):1133–8. doi: 10.5958/2395-146x.2021.00158.7

[80] Moya-Cuevas J, Pérez-Alonso M-M, Ortiz-García P, Pollmann S. Beyond the usual suspects: physiological roles of the arabidopsis amidase signature (AS) superfamily members in plant growth processes and stress responses. Biomolecules. 2021;11(8):1207. doi: 10.3390/biom11081207 34439873 PMC8393822

[81] García I, Castellano JM, Vioque B, Solano R, Gotor C, Romero LC. Mitochondrial beta-cyanoalanine synthase is essential for root hair formation in Arabidopsis thaliana. Plant Cell. 2010;22(10):3268–79. doi: 10.1105/tpc.110.076828 20935247 PMC2990132

[82] Lee HY, Chen Z, Zhang C, Yoon GM. Editing of the OsACS locus alters phosphate deficiency-induced adaptive responses in rice seedlings. J Exp Bot. 2019;70(6):1927–40. doi: 10.1093/jxb/erz074 30810167 PMC6436150

[83] Yin L, Zhang X, Gao A, Cao M, Yang D, An K, et al. Genome-wide identification and expression Analysis of 1-Aminocyclopropane-1-Carboxylate Synthase (ACS) Gene Family in Chenopodium quinoa. Plants (Basel). 2023;12(23):4021. doi: 10.3390/plants12234021 38068656 PMC10707884

[84] Hirose N, Takei K, Kuroha T, Kamada-Nobusada T, Hayashi H, Sakakibara H. Regulation of cytokinin biosynthesis, compartmentalization and translocation. J Exp Bot. 2008;59(1):75–83. doi: 10.1093/jxb/erm157 17872922

[85] Li S-M, Zheng H-X, Zhang X-S, Sui N. Cytokinins as central regulators during plant growth and stress response. Plant Cell Rep. 2021;40(2):271–82. doi: 10.1007/s00299-020-02612-1 33025178

[86] Wang Q, Zhu Y, Zou X, Li F, Zhang J, Kang Z, et al. Nitrogen deficiency-induced decrease in cytokinins content promotes rice seminal root growth by promoting root meristem cell proliferation and cell elongation. Cells. 2020;9(4):916. doi: 10.3390/cells9040916 32283600 PMC7226747

[87] Sun X, Gu Y, Liu Y, Liu Z, Wang P. Nitrogen-driven orchestration of lateral root development: molecular mechanisms and systemic integration. Biology (Basel). 2025;14(8):1099. doi: 10.3390/biology14081099 40906392 PMC12383557

[88] Sakakibara H. Nitrate-specific and cytokinin-mediated nitrogen signaling pathways in plants. J Plant Res. 2003;116(3):253–7. doi: 10.1007/s10265-003-0097-3 12836045

[89] Sultana N, Islam S, Juhasz A, Yang R, She M, Alhabbar Z, et al. Transcriptomic study for identification of major nitrogen stress responsive genes in australian bread wheat cultivars. Front Genet. 2020;11:583785. doi: 10.3389/fgene.2020.583785 33193713 PMC7554635

[90] Sharma N, Sinha VB, Prem Kumar NA, Subrahmanyam D, Neeraja CN, Kuchi S, et al. Nitrogen use efficiency phenotype and associated genes: roles of germination, flowering, root/shoot length and biomass. Front Plant Sci. 2021;11:587464. doi: 10.3389/fpls.2020.587464 33552094 PMC7855041

[91] Sathee L, Jagadhesan B, Pandesha PH, Barman D, Adavi B S, Nagar S, et al. Genome editing targets for improving nutrient use efficiency and nutrient stress adaptation. Front Genet. 2022;13:900897. doi: 10.3389/fgene.2022.900897 35774509 PMC9237392

[92] Watanabe K, Peloquin SJ. Occurrence of 2n pollen and ps gene frequencies in cultivated groups and their related wild species in tuber-bearing Solanums. Theor Appl Genet. 1989;78(3):329–36. doi: 10.1007/BF00265292 24227237

[93] McHale NA. Environmental induction of high frequency 2n pollen formation in diploid Solanum. Can J Genet Cytol. 1983;25(6):609–15. doi: 10.1139/g83-091

[94] Watanabe K. Potato genetics, genomics, and applications. Breed Sci. 2015;65(1):53–68. doi: 10.1270/jsbbs.65.53 25931980 PMC4374564

[95] Watanabe K, El-Nashaar HM, Iwanaga M. Transmission of bacterial wilt resistance by First Division Restitution (FDR) 2n pollen via 4x×2x crosses in potatoes. Euphytica. 1992;60(1):21–6. doi: 10.1007/bf00022254

