# Supplementary material for: Exploring the genetic factors of nitrogen use efficiency in potato
Source: PLoS One. 2025 Nov 14;20(11):e0325578. doi: 10.1371/journal.pone.0325578 (PMC12617955; doi:10.1371/journal.pone.0325578)
Supplement: S3 Table — (DOCX) [file pone.0325578.s003.docx]

Table S2. ANOVA of the variables evaluated under high and low N conditions

|  | | | | |  |  | | | | |
| --- | --- | --- | --- | --- | --- | --- | --- | --- | --- | --- |
| **Spad 74 DAP** | |  |  |  |  | **Stem (count)** | |  |  |  |
| Source | Df | Sum Sq | F value | Pr(>F) |  | Source | Df | Sum Sq | F value | Pr(>F) |
| Genotype | 98 | 7765 | 4.29 | <2e-16 |  | Genotype | 91 | 632 | 3.78 | <2e-16 |
| Treatment | 1 | 5947 | 321.80 | <2e-16 |  | Treatment | 1 | 24 | 13.07 | 0.00033 |
| G x T | 98 | 2869 | 1.58 | 0.00096 |  | G x T | 91 | 219 | 1.31 | 0.03898 |
| Replication | 3 | 107 | 1.94 | 0.12293 |  | Replication | 3 | 4 | 0.77 | 0.5118 |
| Residuals | 4.65 | 8594 |  |  |  | Residuals | 470 | 863 |  |  |
| **%C Aerial** | |  |  |  |  | **%N Tuber** | |  |  |  |
| Source | Df | Sum Sq | F value | Pr(>F) |  | Source | Df | Sum Sq | F value | Pr(>F) |
| Genotype | 91 | 2554 | 6.03 | <2e-16 |  | Genotype | 90 | 146 | 9.97 | <2e-16 |
| Treatment | 1 | 78 | 1.6e1 | 5.5e-5 |  | Treatment | 1 | 9 | 53.89 | 1.5e-12 |
| G x T | 91 | 445 | 1.05 | 0.370 |  | G x T | 90 | 30 | 2.01 | 3.6e-6 |
| Replication | 3 | 44 | 3.1 | 0.025 |  | Replication | 3 | 0 | 0.24 | 0.87 |
| Residuals | 359 | 1671 |  |  |  | Residuals | 352 | 57 |  |  |
| **AgNUE** | |  |  |  |  | **NUE** | |  |  |  |
| Source | Df | Sum Sq | F value | Pr(>F) |  | Source | Df | Sum Sq | F value | Pr(>F) |
| Genotype | 91 | 183.072 | 3142.74 | < 2e-16 |  | Genotype | 71 | 7486 | 1618 | <2e-16 |
| Treatment | 1 | 822 | 5.07 | 0.025 |  | Treatment | 1 | 16 | 1.45 | 0.229 |
| G x T | 91 | 43373 | 2.94 | 1.9e-13 |  | G x T | 71 | 1329 | 1.74 | 0.00066 |
| Replication | 3 | 41 | 0.08 | 0.969 |  | Replication | 3 | 32 | 1.01 | 0.39 |
| Residuals | 396 | 64258 |  |  |  | Residuals | 329 | 3534 |  |  |
| **HI** | |  |  |  |  | **Stem Length** | |  |  |  |
| Source | Df | Sum Sq | F value | Pr(>F) |  | Source | Df | Sum Sq | F value | Pr(>F) |
| Genotype | 86 | 10.6 | 5.85 | <2e-16 |  | Genotype | 85 | 124293 | 11.87 | <2e-16 |
| Treatment | 1 | 1.4 | 64.85 | 7.6e-15 |  | Treatment | 1 | 131700 | 1068.89 | <2e-16 |
| G x T | 86 | 1.8 | 0.98 | 0.54 |  | G x T | 85 | 44022 | 4.20 | <2e-16 |
| Replication | 3 | 0.0 | 0.76 | 0.52 |  | Replication | 3 | 922 | 2.49 | 0.059 |
| Residuals | 441 | 9.3 |  |  |  | Residuals | 423 | 52119 |  |  |
|  |  |  |  |  |  |  |  |  |  |  |
| **Tuber (count)** | |  |  |  |  |  |  |  |  |  |
| Source | Df | Sum Sq | F value | Pr(>F) |  |  |  |  |  |  |
| Genotype | 93 | 5458 | 9.86 | <2e-16 |  |  |  |  |  |  |
| Treatment | 1 | 2687 | 451.58 | <2e-16 |  |  |  |  |  |  |
| G x T | 93 | 2454 | 4.43 | <2e-16 |  |  |  |  |  |  |
| Replication | 3 | 6 | 0.32 | 0.81 |  |  |  |  |  |  |
| Residuals | 443 | 2636 |  |  |  |  |  |  |  |  |
|  |  |  |  |  |  |  |  |  |  |  |
|  |  |  |  |  |  |  |  |  |  |  |
|  |  |  |  |  |  |  |  |  |  |  |
|  |  |  |  |  |  |  |  |  |  |  |
|  |  |  |  |  |  |  |  |  |  |  |
|  |  |  |  |  |  |  |  |  |  |  |
|  |  |  |  |  |  |  |  |  |  |  |
|  |  |  |  |  |  |  |  |  |  |  |
|  |  |  |  |  |  |  |  |  |  |  |
|  |  |  |  |  |  |  |  |  |  |  |
|  |  |  |  |  |  |  |  |  |  |  |
|  |  |  |  |  |  |  |  |  |  |  |
|  |  |  |  |  |  |  |  |  |  |  |
|  |  |  |  |  |  |  |  |  |  |  |
|  |  |  |  |  |  |  |  |  |  |  |
|  |  |  |  |  |  |  |  |  |  |  |
|  |  |  |  |  |  |  |  |  |  |  |
|  |  |  |  |  |  |  |  |  |  |  |
|  |  |  |  |  |  |  |  |  |  |  |
|  |  |  |  |  |  |  |  |  |  |  |
|  |  |  |  |  |  |  |  |  |  |  |
|  |  |  |  |  |  |  |  |  |  |  |
|  |  |  |  |  |  |  |  |  |  |  |
|  |  |  |  |  |  |  |  |  |  |  |
|  |  |  |  |  |  |  |  |  |  |  |
|  |  |  |  |  |  |  |  |  |  |  |
|  |  |  |  |  |  |  |  |  |  |  |
|  |  |  |  |  |  |  |  |  |  |  |
|  |  |  |  |  |  |  |  |  |  |  |
|  |  |  |  |  |  |  |  |  |  |  |
|  |  |  |  |  |  |  |  |  |  |  |
|  |  |  |  |  |  |  |  |  |  |  |
|  |  |  |  |  |  |  |  |  |  |  |
|  |  |  |  |  |  |  |  |  |  |  |
|  |  |  |  |  |  |  | |  |  |  |
|  |  |  |  |  |  |  |  |  |  |  |
|  |  |  |  |  |  |  |  |  |  |  |
|  |  |  |  |  |  |  |  |  |  |  |
|  |  |  |  |  |  |  |  |  |  |  |
|  |  |  |  |  |  |  | |  |  |  |
|  |  |  |  |  |  |  |  |  |  |  |
|  |  |  |  |  |  |  |  |  |  |  |
|  |  |  |  |  |  |  |  |  |  |  |
|  |  |  |  |  |  |  |  |  |  |  |
|  |  |  |  |  |  |  |  |  |  |  |
|  |  |  |  |  |  |  |  |  |  |  |
|  |  |  |  |  |  |  |  |  |  |  |
|  |  |  |  |  |  |  |  |  |  |  |
| Residuals | 212.00 | 3.71 |  |  |  | Residuals | 232.00 | 5.72 |  |  |
|  |  |  |  |  |  |  |  |  |  |  |
|  |  |  |  |  |  |  |  |  |  |  |
|  |  |  |  |  |  |  |  |  |  |  |
|  |  |  |  |  |  |  |  |  |  |  |
|  |  |  |  |  |  |  |  |  |  |  |
|  |  |  |  |  |  |  |  |  |  |  |
|  |  |  |  |  |  |  |  |  |  |  |
|  |  |  |  |  |  |  |  |  |  |  |
|  |  |  |  |  |  |  |  |  |  |  |
|  |  |  |  |  |  |  |  |  |  |  |
|  |  |  |  |  |  |  |  |  |  |  |
|  |  |  |  |  |  |  |  |  |  |  |
|  |  |  |  |  |  |  |  |  |  |  |
|  |  |  |  |  |  |  |  |  |  |  |
|  |  |  |  |  |  |  |  |  |  |  |
|  |  |  |  |  |  |  |  |  |  |  |
|  |  |  |  |  |  |  |  |  |  |  |
|  |  |  |  |  |  |  |  |  |  |  |
|  |  |  |  |  |  |  |  |  |  |  |
|  |  |  |  |  |  |  |  |  |  |  |
|  |  |  |  |  |  |  |  |  |  |  |
|  |  |  |  |  |  |  |  |  |  |  |
|  |  |  |  |  |  |  |  |  |  |  |
|  |  |  |  |  |  |  |  |  |  |  |
|  |  |  |  |  |  |  |  |  |  |  |
|  |  |  |  |  |  |  |  |  |  |  |
|  |  |  |  |  |  |  |  |  |  |  |
|  |  |  |  |  |  |  |  |  |  |  |
|  |  |  |  |  |  |  |  |  |  |  |
|  |  |  |  |  |  |  |  |  |  |  |
|  |  |  |  |  |  |  |  |  |  |  |
|  |  |  |  |  |  |  |  |  |  |  |
|  |  |  |  |  |  |  |  |  |  |  |
|  |  |  |  |  |  |  |  |  |  |  |
|  |  |  |  |  |  |  |  |  |  |  |
|  |  |  |  |  |  |  |  |  |  |  |
|  |  |  |  |  |  |  |  |  |  |  |
|  |  |  |  |  |  |  |  |  |  |  |
|  |  |  |  |  |  |  |  |  |  |  |
|  |  |  |  |  |  |  |  |  |  |  |
