# Supplementary figures and images for: Exploring the genetic factors of nitrogen use efficiency in potato
Source: PLoS One. 2025 Nov 14;20(11):e0325578. doi: 10.1371/journal.pone.0325578 (PMC12617955; doi:10.1371/journal.pone.0325578)

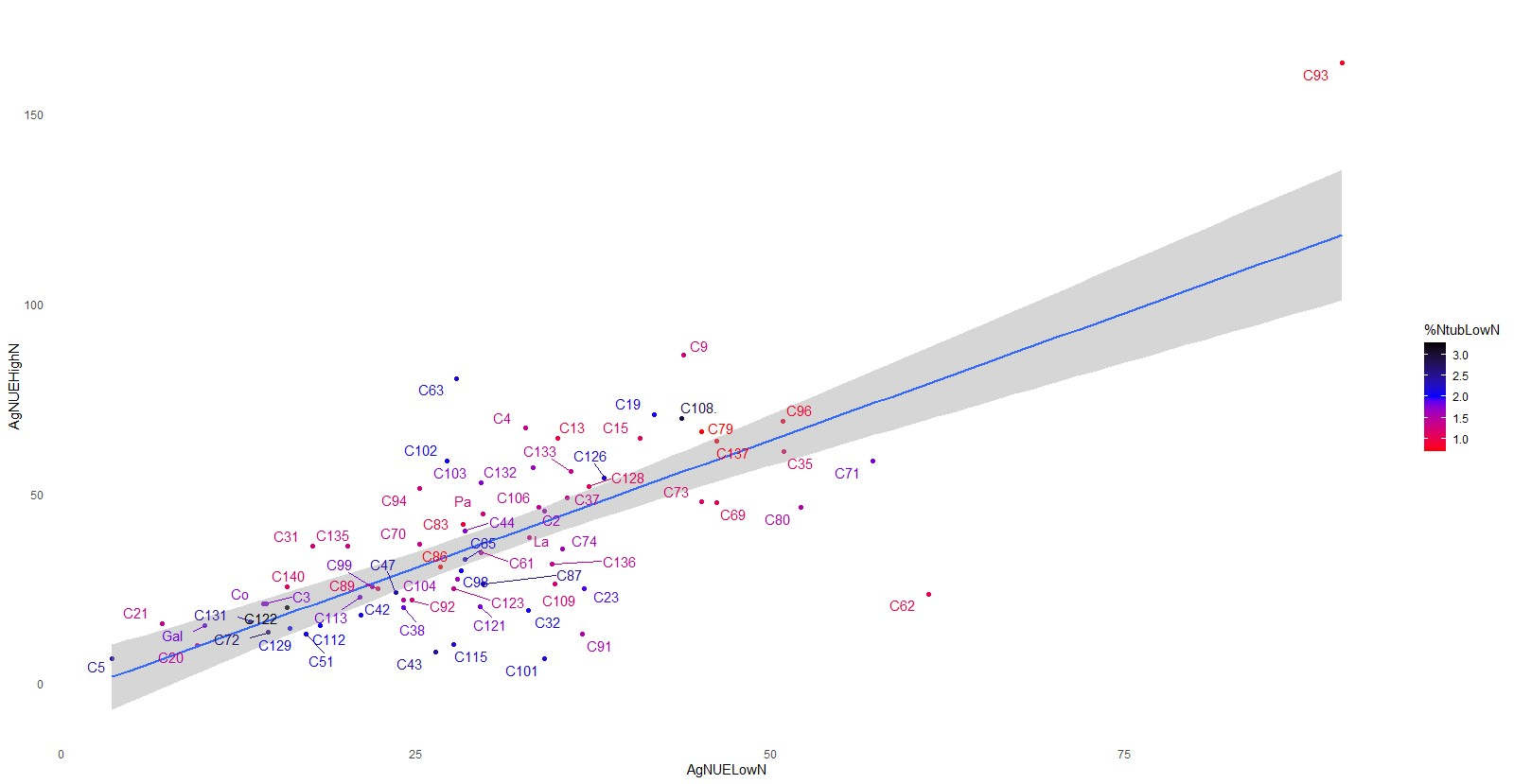

Supplement: S1 Fig — Each point represents a genotype. The color gradient indicates the percentage of nitrogen accumulated in tubers under low nitrogen conditions (%NtubLowN), with green tones indicating lower values and darker tones higher values. (TIFF) [file pone.0325578.s008.tiff]

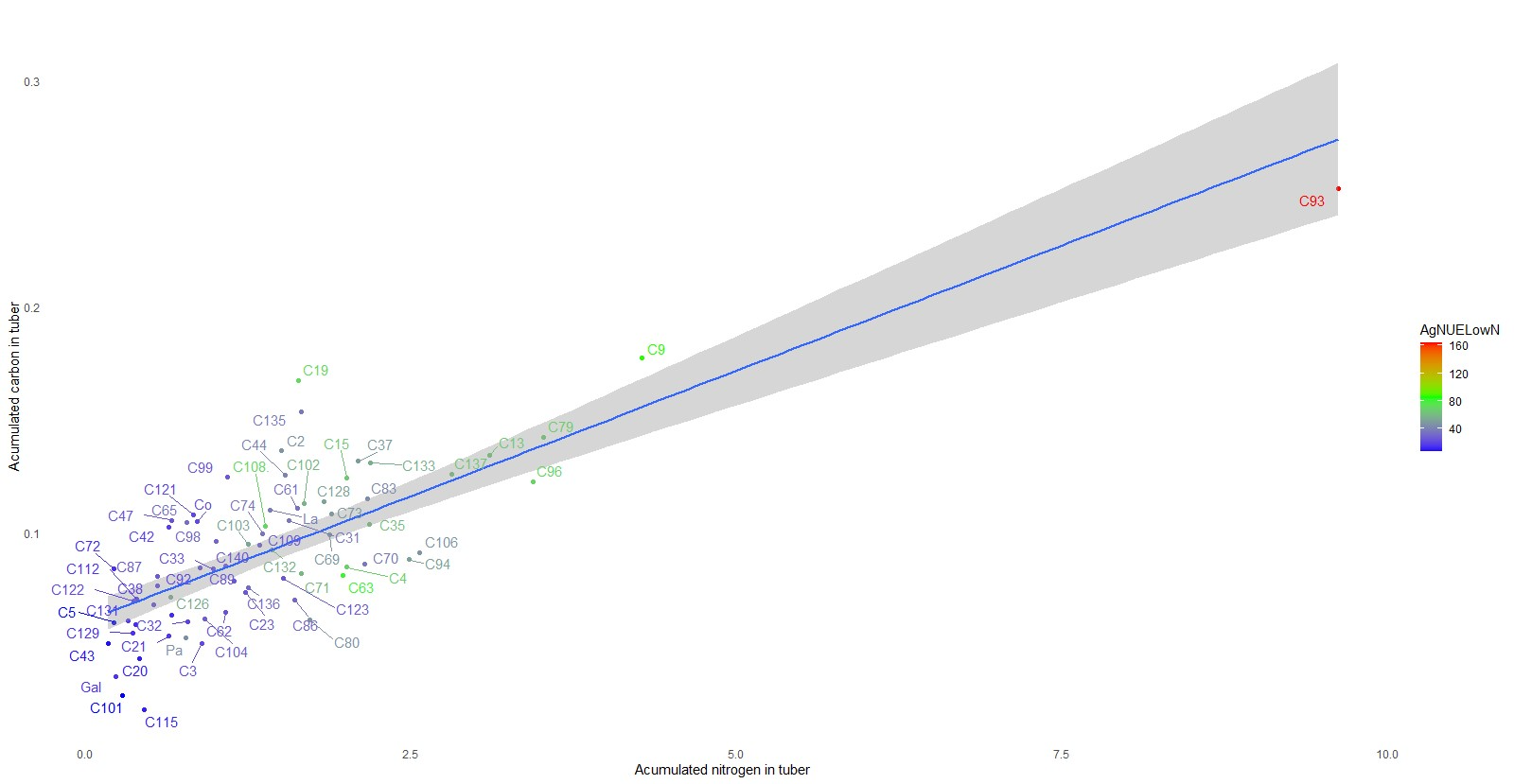

Supplement: S2 Fig — Each point represents a genotype. The color gradient indicates Agronomic Nitrogen Use Efficiency (AgNUE), with green tones representing lower values and darker tones higher values. (TIFF) [file pone.0325578.s009.tiff]

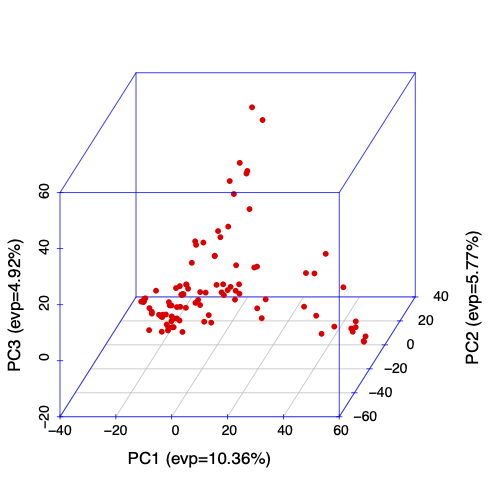

Supplement: S3 Fig — (TIFF) [file pone.0325578.s010.tiff]

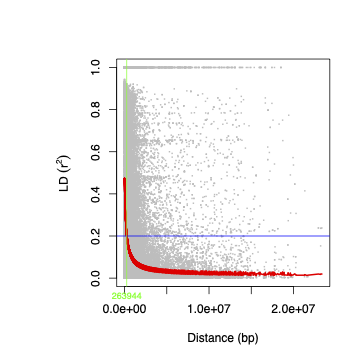

Supplement: S4 Fig — (TIFF) [file pone.0325578.s011.tiff]
